# Is Photocatalysis the Next Technology to Produce Green Hydrogen to Enable the Net Zero Emissions Goal?

**DOI:** 10.1002/gch2.202200165

**Published:** 2022-12-16

**Authors:** Mark Isaacs, Julio Garcia‐Navarro, Wee‐Jun Ong, Pablo Jiménez‐Calvo

**Affiliations:** ^1^ Department of Chemistry University College London 20 Gower Street London WC1H 0AJ UK; ^2^ HarwellXPS Research Complex at Harwell Rutherford Appleton Lab Didcot OX11 0FA UK; ^3^ Stichting New Energy Coalition Nijenborgh 6 Groningen 9747 AG The Netherlands; ^4^ School of Energy and Chemical Engineering Xiamen University Malaysia Selangor Darul Ehsan Selangor 43900 Malaysia; ^5^ Center of Excellence for NaNo Energy & Catalysis Technology (CONNECT) Xiamen University Malaysia Selangor Darul Ehsan Selangor 43900 Malaysia; ^6^ State Key Laboratory of Physical Chemistry of Solid Surfaces College of Chemistry and Chemical Engineering Xiamen University Xiamen 361005 P. R. China; ^7^ Shenzhen Research Institute of Xiamen University Shenzhen 518057 P. R. China; ^8^ Department of Colloid ChemistryMax‐Planck‐Institute of Colloids and InterfacesAm Mühlenberg 1 14476 Potsdam Germany

**Keywords:** hydrogen, net zero emissions, photocatalysis, roadmap, technological development

## Abstract

Energy security concerns require novel greener and more sustainable processes, and Paris Agreement goals have put in motion several measures aligned with the 2050 roadmap strategies and net zero emission goals. Renewable energies are a promising alternative to existing infrastructures, with solar energy one of the most appealing due to its use of the overabundant natural source of energy. Photocatalysis as a simple heterogeneous surface catalytic reaction is well placed to enter the realm of scaling up processes for wide scale implementation. Inspired by natural photosynthesis, artificial water splitting's beauty lies in its simplicity, requiring only light, a catalyst, and water. The bottlenecks to producing a high volume of hydrogen  are several: Reactors with efficient photonic/mass/heat profiles, multifunctional efficient solar‐driven catalysts, and proliferation of pilot devices. Three case studies, developed in Japan, Spain, and France are showcased to emphasize efforts on a pilot and large‐scale examples. In order for solar‐assisted photocatalytic H_2_ to mature as a solution, the aforementioned bottlenecks must be overcome for the field to advance its technology readiness level, assess the capital expenditure, and enter the market.

## Graphical abstract

The 2050 roadmap strategies and net zero emission goals are made possible through the adoption of novel greener and more sustainable processes. In this regard, exploiting solar irradiation (a plentiful energy source) is advantageous for developing green hydrogen technologies. Photocatalytic devices are suited for large‐scale plants proliferation. Three case studies from France, Spain, and Japan are presented.

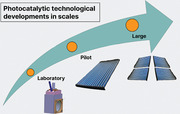

## 1 Introduction

Today, humankind is confronted by a series of global challenges: energy/environmental crises, population growth, pandemics, and geopolitical wars, that simultaneously promote the depletion of natural resources and accelerate the contamination of sources of life: water, air, and soil.^[^ ^1^ ^]^ Among these challenges, energy production, and demand deserve special attention by the research and development communities who urgently need to devise tangible solutions. Notably, the continuous conversion of fossil fuels, such as oil, gas, and coal continue to contribute to greenhouse gases emissions, such as carbon dioxide (CO_2_).^[^ ^2^ ^]^ Ideally, certain criteria for selecting the future energy sources should be observed, namely, these sources should be abundant, easy to recycle or regenerate at the large scale, and their conversion should be CO_2_‐free.

**Figure 1:**
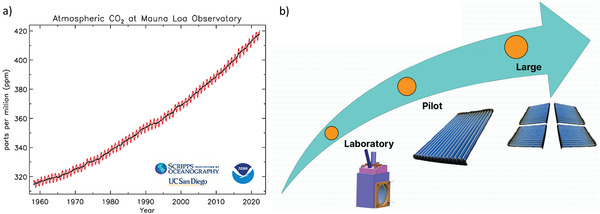
a) Measurements of atmospheric CO_2_ since 1958 from the Mauna Loa Observatory in Hawaii (black) and from the South Pole (red) show a steady annual increase in atmospheric CO_2_ concentration. Reproduced with permission.^[^ ^5^ ^]^ Copyright 2022, Scripps CO_2_ Program. b) Illustrative scheme to represent the technological transfer from laboratory setups, pilot scale devices, and large‐plants.

The world's total net CO_2_ emissions, from 1850 to 2019, were 2400 ± 240 Gt(CO_2_). Between 2010 and 2019 alone, roughly 17% of the total cumulative net CO_2_ emissions (410 ± 30 GtCO_2_) were released,^[^ ^3^ ^]^ supporting Hubbert's predictions from 1970.^[^ ^4^ ^]^ The concentration of CO_2_ in the atmosphere has been directly measured by the Mauna Loa Observatory, exhibiting ≈417 ppm **Figure** **1**.^[^ ^5^ ^]^ Such CO_2_ increases result in damning and imminent consequences to the natural world.^[^ ^6^ ^]^ For example, increases to the surface temperature of the Earth consequently impact the average rate of sea levels (e.g., 20 cm rise between 1901 and 2018), desert dryness, melting of glaciers and ice caps, heat content and acidification of the ocean, and other negative effects to various ecosystems.^[^ ^7^ ^]^

For these reasons, an assessment of existing processes must be carried out to replace them with more efficient and sustainable technologies. For that, a shift from fossil fuels to clean energy has been suggested by the roadmap 2050 (the European Commission (EU))^[^ ^8^ ^]^ and Net Zero by 2050 (the EU Green Deal and International Energy Agency).^[^ ^9^, ^10^ ^]^ At the present time, fossil fuels still supply 80% of the world's energy.

In this context, the use of solar energy is an appealing alternative due to the primary energy source being an effectively unlimited supply of great power.^[^ ^11^, ^12^ ^]^ The diversity of emitted photons with different energies that can be selectively captured and thus used for chemical conversion to obtain solar clean fuels makes this a unique source.^[^ ^13^ ^]^ Nevertheless, the capture of solar photons is a challenge due to the day‐night cycle, seasonal change, cloud presence, and geographical local distribution.^[^ ^14^ ^]^

Among several solar‐driven fuels currently under investigation, hydrogen is primarily unlocking the potential of renewables as an energy source, and moving carbon neutrality toward a reality rather than a myth.^[^ ^15^ ^]^ The attractiveness of H_2_, as an energy vector, lies in three arguments: a) Its high energy content per unit mass (≈142 kJ mol^−1^), twofold to threefold increase compared with classical fossil fuels, such as gasoline, methane, propane, and natural gas,^[^ ^16^ ^]^ b) versatile energy storage medium because it can be used in electric only or combined heat and power fuel cells in stationary applications, internal combustion engines, and fuel cell vehicles,^[^ ^17^ ^]^ and c) despite its low self‐discharge rate, regenerative hydrogen fuel cells may be cheaper than batteries in an optimized energy arbitrage system.^[^ ^18^ ^]^

H_2_ integration into the economic model became a serious consideration following the signing of the Paris agreement by the members of United Nations (UN).^[^ ^19^ ^]^ One of the goals of this agreement is to decarbonize heavy industry and, for that, reinviting or shifting existing technologies for optimized, greener, and more sustainable processes is a must. Thus, the European Commission, the World Economic Forum, the UN, Mission Innovation, and established industries are implementing new strategies to support net zero emissions and update their portfolio of technologies. With this integration scenario, emerging H_2_ technologies are achieving historical technology readiness levels (TRL), ranging from laboratory setups into pilot devices and large‐plants (Figure 1b). However, storage, transport, and safety hindrances must be addressed and refined to enable fully widespread integration into the socio‐economical context.^[^ ^16^ ^]^

H_2_ safety is undoubtedly a topic to be addressed for a broader implementation and social acceptance.^[^ ^20^ ^]^ Briefly, the ignition energy of H_2_ mixed with air (0.017 mJ) is lower than natural gas (0.24–0.31 mJ).^[^ ^21^ ^]^ As natural gas is already handle in a large scale making a proof‐of‐concept. Thus, the ignition energy difference points out that theoretically H_2_ would cause lower flame propagation from the ignition point, thus is safer. Therefore, an ignition endanger will be consider when a source is close (e.g., spark or over heated contact). Experiments have shown that H_2_ does not ignite in cases where it was expected under specific circumstances, namely in presence of friction or energized devices.^[^ ^21^ ^]^ Still, more maturity in ignition tests is needed to mitigate and minimize inherent risks (essentially ignition), as reached for natural gas.

## 2 Hydrogen Production Context

Worldwide political and scientific consensus have defined a color nomenclature for H_2_ in function of its source of production. There are eight different colors but for this article we limit the introduction of the three extreme cases, from the most and least contaminant and neutral sources. Please refer to Ajanovic et al. for details.^[^ ^22^ ^]^ Grey H_2_ is made with fossil‐fuel‐based technology, blue H_2_ is climate‐neutral and made with carbon capture and storage technology, and green H_2_ is made with renewable energy (using renewables).^[^ ^23^ ^]^

To date, H_2_ is produced by mostly fossil fuels (98%) emitting nearly 900 million metric tons (Mt) of CO_2_ per year.^[^ ^24^ ^]^ By 2020 H_2_ production reached 90 Mt.^[^ ^24^, ^25^ ^]^ It is forecasted, however, that H_2_ production may surpass 200 Mt in 2030. Steam methane reforming (76%) is the leading H_2_ production technology in the market, followed by coal gasification (22%), two energy‐intensive and highly polluting methods.^[^ ^26^ ^]^ Electrolysis, a clean but relatively adolescent technology^[^ ^25^ ^]^ is already in the loop of existing technological processes contributing to 2% of total production, evidencing the need to decentralize H_2_ production to facilitate the integration with downstream processes to foster a sustainable energy transition.

The output stream of electrolyzers is a mixture of H_2_ and O_2_.^[^ ^27^ ^]^ Thus, a common practice consists in using gas separation processes, (also in fuel cells and steam methane reforming with the equivalent or other by‐product gases) in the end production point with possible recirculation for increasing the efficiency itself and obtained a higher H_2_ purity. The high‐quality H_2_ with the desired low impurity levels is achievable with either adsorption and diffusion purification processes.^[^ ^28^ ^]^ The commercial technology widely used for adsorption‐based H_2_ purification is Pressure Swing Adsorption (PSA), where porous solid adsorbents (e.g., zeolites and activated carbons) preferentially adsorb particular components from a gas stream in a pressurized vessel, later to be released after depressurization, that is, pressure cycles.^[^ ^29^ ^]^ Besides PSA, there are other methods based on temperature (TSA) and/or vacuum (VSA) but purities and cost differ.^[^ ^30^ ^]^

The amount of H_2_ made from renewable sources, like water or biomass, is a small part of the total amount produced.^[^ ^31^ ^]^ Fortunately, there are several methods to obtained H_2_ and O_2_ via water splitting (WS), namely electrolysis, electrocatalysis, photocatalysis (PC), photoelectrocatalysis, photovoltaic‐electrochemical, solar thermochemical cycles, photothermal catalytic, or photobiological processes.^[^ ^32^ ^]^

WS is an uphill reaction that requires a significant Gibbs free energy, that is, 237 kJ mol^−1^.^[^ ^33^ ^]^ Interestingly, WS requires a very similar amount of energy as natural photosynthesis, with plants needing 1.24 eV (energy required per electron driven through the photosynthetic system) to make glucose,^[^ ^34^ ^]^ whereas to split water artificially requires 1.23 eV per electron.^[^ ^35^ ^]^ This thermodynamic requirement of PC H_2_ production is defined primarily by the water oxidation (oxygen evolution reaction) and then the proton reduction (hydrogen evolution reaction, HER) half‐reactions requiring 1.23 and 0 V versus normal hydrogen electrode (pH = 0), respectively.

On top of H_2_ production via WS, PC is an appealing approach for other energy applications, such as CO_2_ reduction and nitrogen fixation.^[^ ^36^ ^]^ As heterogeneous catalysis type, PC is particularly simple,^[^ ^37^ ^]^ requiring only light to activate the solid semiconductor (SC) and transform either a liquid or gas reactant.^[^ ^38^ ^]^ Unlike, typical electrolyzer connected with a photovoltaic panel, PC simplifies in its unassisted electricity possibility, potentially lowering costs in an operating expenses (OPEX) viewpoint.

## 3 Photocatalytic Water Splitting Principle

PC WS is a heterogeneous surface catalytic reaction and possesses an attractive simplicity, requiring only light, a catalyst, and water to function. For that reason, PC WS is an appealing prospect, and considered one of the “holy grail” reactions in physical chemistry because can directly produce H_2_ from water. Should the technical barriers be removed whilst maintaining even a moderate capital expenditure (CAPEX), then one can presume it to challenge competitor methods, perhaps eventually reaching an even more attractive cost.

**Figure 2:**
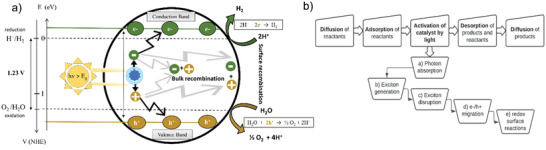
a) Photocatalyst scheme of activation, generation, and reactional water splitting steps. b) Summary of general photocatalytic steps.

Typically, PC WS starts (**Figure** **2**a) when a SC is irradiated with a photon with equal or higher energy than its band gap. This generates an exciton: excited electron (e^−^) and a positive hole (h^+^). These two charge carriers dissociate and migrate to the surface of the catalyst. Some e^−^/h^+^ partners face undesirable recombination on the surface or in the bulk, but the e^−^/h^+^ pairs reaching the surface may undertake one of the two WS half‐reactions.

Successful h^+^ moieties avoiding recombination can oxidize water to create O_2_ and H^+^, while on the other hand, available e^−^ species can reduce the H^+^ to obtain H_2_. Therefore, the HER consumes 2 e^−^ per molecule of H_2_ formed.^[^ ^35^ ^]^ The four steps of the process (activation, exciton formation, recombination, redox) all involve different time scales, which are well‐described by Takanabe et al.^[^ ^35^ ^]^

Figure 2b summarizes PC WS sequential steps: a) Activation of the catalyst (critical), b) generation of the charge carriers, and c) redox reactions in the interface between the catalyst and the substrate.^[^ ^39^ ^]^ Prior to catalyst activation there are two pre‐reaction steps, first the reactant molecules must diffuse to the liquid‐gas/solid interface to be adsorbed onto the catalyst surface. Once on the surface, they then wait to encounter a photo‐generated charge carrier and react. Furthermore, after the catalyst's activation, two analogous post‐reaction steps then happen. They initially desorb from the catalyst surface and then diffuse back into the reaction media.

The critical step (activation of the catalyst) defines the overall efficacy of the material, which occurs mostly in the interior of the catalyst, but some sub‐steps may proceed at its surface. Many leading scientists are currently improving the reaction medium/catalyst interface contact with sophisticated advanced material strategies (Z‐scheme,^[^ ^40^ ^]^ Schottky^[^ ^41^, ^42^ ^]^ junctions, and co‐polymerization,^[^ ^43^ ^]^ among others) to tune key properties and increase efficiencies.

## 4 Photocatalytic Technological Developments

This section will showcase three photocatalytic systems develop in Asia and Europe. One recently automated laboratory scale reactor, and two pilot devices: A panel photocatalytic array and a compound parabolic collector (CPC). The reasoning is to highlight the international efforts toward improving laboratory photocatalytic systems and the transition into pilot and large‐scale processes. The first step toward large‐plants, however, is the development and refinement of effective PC devices capable of promising performance on the pilot scale.

Considerations of the complexities of benchmarking and effective engineering challenges in developing lab‐scale PC materials was addressed in our recent work designing and constructing a compact automated stainless steel reactor, of 40 cm^3^ of volume.^[^ ^41^ ^]^ H_2_ photoproduction and quantum yields exhibited onefold‐ to twofold increases when compared to literature equivalent materials. This result emphasizes that reactor geometry and configuration setup play a key role in the performance of PC materials and reveals insights into ideal (minimal losses) heat/mass/photonic profiles.

An unprecedented 100 m^2^ arrayed panel system led by Professor Domen, comprising 1600 units has been recently launched at Kakioka Research Facility at the University of Tokyo.^[^ ^44^ ^]^ This system design has achieved 0.76% solar‐to‐hydrogen (STH) conversion, similar to ideal lab scale efficiency, ≈1% STH. The configuration attractiveness relies in being simple, cheaper, and more amenable to scale‐up compared with solar cells and/or electrolysis systems. Each panel plate was sprayed with a modified aluminum‐doped strontium titanate photocatalyst layer, one of the most efficient photocatalysts to date.^[^ ^45^ ^]^ The highlight of this panel array is its H_2_ recovery after several months of continuous operationality, starting with a moist gas product mixture, and H_2_ capture with a polyimide membrane.

Another pilot device for solar H_2_ generation and removal of wastewater pollutants was tested at Plataforma Solar de Almeria.^[^ ^46^ ^]^ This setup consists of a CPC, which is a reactor type enabling highly efficient solar photon collection. Two materials were tested, Pt/(TiO_2_‐N) and Pt/(CdS‐ZnS), with the former outperforming the latter in combination with two sacrificial electron donors: formic acid and glycerol. This system evidences other proof‐of‐concept of H_2_ production, though this time using municipal wastewaters, enabling simultaneous waste water depollution and energy vector generation.

Though several photocatalytic WS prototypes are available, their efficiency is still low (<1%). From a photochemical process perspective, reactor design and process optimization are needed to make this technology viable and feasible on a relevant scale. Furthermore, from a materials science perspective, higher‐performing photocatalysts with better stability and H_2_ production efficiency (5–10% STH) are needed for economic viability.

Public and private partnerships are proliferating internationally to tackle these bottlenecks; The Green Deal (European Union) in particular is investing a significant amount into finding tangible renewable solutions.

## 5 Conclusions and Outlook

By maturing solar‐assisted photocatalytic H_2_ technologies on a pilot scale, like in the three case studies, the proof‐of‐concept stage can be bypassed and thus enable the new chapter of TRL acceleration. The innovation of advanced H_2_ pilot devices in the next half‐decade will be key to unlocking new engineering advances for large‐scale that may foster both the commercialization of H_2_ from solar‐fueled photocatalysis across the global market and the circular economy (while decarbonizing with clean energy).

For solar scale up reactor design and dimensional technological transfer analysis, we propose a (non‐exhaustive) list of parameters extracted from the selected case studies to implement in higher TRL projects, essentially in four axes: operational, photonic, mass, and heat transfer profiles. We suggest such considerations with the intention that WS PC pilot devices proliferate and unlock further technological barriers.

Operational considerations include quantification of H_2_ (data acquisition) within an integrated on‐line analytical equipment (gas chromatography) to reduce operational costs in human resources—which is essential for scaling up to TRL ≥3 (pilot prototypes). Reactor components should have resistance to pH, corrosion, and exposed environmental conditions. Furthermore, components must be easy to handle and accessible (with a modular assembly design providing an optimal solution to this point), and finally low‐cost (low CAPEX and OPEX). Photonic considerations for catalyst activation focus on efficient photon capture and distribution, with suitable geometry to maximize collected light, suitable light absorbing materials, and maximizing use of the solar spectrum. Mass transfer considerations facilitate adsorption–desorption interactions, for example the reactor must guarantee minimal pressure from the gas flow, ensure suspended particle homogeneity in the contained volume, and avoid creation of pronounce vortexes of sufficient agitation to disrupt processes. Heat transfer considerations include the use of recirculating chillers directly in the path of the light source or inside the reactor (but not in contact with the particle dissolution) to guarantee a constant temperature, ideally of 20 °C, to avoid thermal catalysis contributions.

Ultimately, for large‐scale setups, the inclusion of wastewater and or organic pollutants as for substrate source and electron or proton donors or so‐called sacrificial agents, respectively, should not be negligible while planning of building the plant. Contrarily, optimal processes envisage the simultaneous reuse of one of source (wastewater)—resulting in the production of decontaminated (of organic pollutants) water and concomitant generation of energy solar carriers.

## Conflict of Interest

The authors declare no conflict of interest.
